# The Lonely Mouse – Single Housing Affects Serotonergic Signaling Integrity Measured by 8-OH-DPAT-Induced Hypothermia in Male Mice

**DOI:** 10.1371/journal.pone.0111065

**Published:** 2014-12-01

**Authors:** Otto Kalliokoski, A. Charlotte Teilmann, Kirsten R. Jacobsen, Klas S. P. Abelson, Jann Hau

**Affiliations:** Department of Experimental Medicine, Faculty of Health and Medical Sciences, University of Copenhagen, Copenhagen, Denmark; Radboud University, Netherlands

## Abstract

Male BALB/c mice single-housed for a period of three weeks were found to respond with a more marked hypothermia to a challenge with a selective serotonergic agonist (8-OH-DPAT) than their group-housed counterparts. This effect of single housing was verified by screening a genetically heterogeneous population of male mice on a C57BL/6 background from a breeding colony. Enhanced activity of the implicated receptor (5-HT_1A_) leading to an amplified hypothermic effect is strongly associated with depressive states. We therefore suggest that the 8-OH-DPAT challenge can be used to demonstrate a negative emotional state brought on by e.g. long-term single housing in male laboratory mice. The study emphasizes the importance of social housing, and demonstrates that male mice deprived of social contact respond with altered serotonergic signaling activity. Male mice not only choose social contact when given the option, as has previously been shown, but will also, when it is deprived, be negatively affected by its absence. We propose that the 8-OH-DPAT challenge constitutes a simple, but powerful, tool capable of manifesting the effect of social deprivation in laboratory mice. It potentially allows not only for an unbiased, biochemical evaluation of psychological stressors, but may also allow for determining whether the effect of these can be counteracted.

## Introduction

A much debated topic in housing laboratory mice is their need for cage mates. Single housing is not uncommon in laboratory animal facilities; experimental design may require it and male mice, in particular, are often housed singly due to their aggression toward other males [1]. How single housing affects mice and the associated studies is however far from fully understood. Mice clearly opt for social contact when presented with a choice – even male mice on the receiving end of male-male aggression will do so [2],[3] – but whether they suffer from the lack of it has been disputed [4]. It has been speculated that the lone mouse may be less capable of coping with external stressors than is its group-housed counterpart [5], but single housing appears, in itself, not to induce an acute stress response [6],[7]. Male mice often respond with aggression to other, unfamiliar males; stable male-male groups are thus preferentially established at weaning [8], often consisting of littermates. Additionally, if isolated for as little as 12–24 h, it has been shown that male mice may become aggressive and territorial, even if re-housed with their littermates [9]. Some studies argue that single-housed mice are no more stressed than group-housed mice [10],[11], effectively suggesting that social isolation may carry no impact on the wellbeing of the mice and the associated studies. There has even been outright advocacy – albeit in the past – of single housing of male mice in the laboratory animal science community [12],[13]. Still, both American [14] and European [15] guidelines today stress the importance of social housing of laboratory mice and there is indirect evidence that single-housed mice experience some form of sub-acute stress [16],[17]. The effect of single housing on laboratory mice is clearly an issue that despite extensive study has yet to be fully understood. The biggest hurdle that must be negotiated is how to identify and quantify the impact brought on by single housing. 8-OH-DPAT is a potent serotonin (5-HT) receptor agonist, preferentially acting on the 5-HT_1A_ receptor [18]. The 5-HT_1A_ receptor is highly implicated as playing an important role in depressive states – from mild anxiety and lowered mood to major depression in many animal species [19],[20] and even suicidal tendencies in humans [21]. Numerous studies into the altered affinities and expression patterns of 5-HT_1A_ in relation to negative stimuli have been carried out [22],[23]. As a simple but crude method for gauging serotonergic signaling integrity, the hypothermic state brought on by 5-HT_1A_ agonists has been studied. The degree of hypothermia has been demonstrated as a well-consolidated indirect measure of affectedness [24],[25].

We propose that hydroxy-dipropylamino-tetralin-(8-OH-DPAT)-induced hypothermia (which we will refer to as HIH) constitutes a simple, but powerful, tool capable of manifesting the effect of social deprivation in laboratory mice. Drawing on previous findings where we noted that single-housed mice belonging to a control group (subjected to no treatment at all) would alter their HIH response over time, seemingly from the circumstances of their housing alone [26], we set out to investigate whether the HIH challenge can be applied in a novel context, evaluating the impact of single housing on male laboratory mice. In addition to a controlled proof-of-concept study we investigated, in a larger opportunistic study, whether single housing of males during routine operations in a mouse breeding unit would also impact serotonergic signaling integrity as evaluated through HIH. The binding affinity of 8-OH-DPAT to 5-HT_1A_ has been shown to increase in socially isolated mice [27],[28], which, in turn, translates to a greater hypothermic response [29]. We thus hypothesized that male mice housed singly would respond with a greater hypothermic response when challenged with 8-OH-DPAT than would their group-housed counterparts.

## Material and Methods

### Housing conditions

All animals were housed in Makrolon cages, provided extruded diet (Altromin 1319; Brogaarden, Gentofte, Denmark) and acidified tap water *ad libitum*. Cages were enriched with a hide, a cardboard tunnel (Lillico, Horley, UK), nest-building material (Lillico), and aspen gnawing sticks on an aspen chip bedding (Tapvei Oy, Kortteinen, Finland). Lighting was kept on a 12∶12 dark/artificial light cycle with lights on from 6.00 A.M and with a 30-min period of reduced lighting at transitions. Temperature was kept at 22°C (±2°C) with a relative humidity of 55%. In Experiment 1 the animals were housed in conventional Eurostandard Type III cages (Tecniplast, Varese, Italy) and provided cardboard shelters (Lillico). In Experiment 2 all animals were housed in either Type II or Type III cages in individually ventilated cage systems (Tecniplast) with 63 h^−1^ air changes and provided with plastic shelters (“JAKO shelter”; Molytex, Glostrup, Denmark). The experiments were carried out in a fully AAALAC (Association for Assessment and Accreditation of Laboratory Animal Care International) accredited facility under the supervision of a local animal welfare committee. The study was approved by the Danish Animal Experiments Inspectorate, carried out under licenses 2011/561-1980 (Experiment 1) and 2012/561-169 (Experiment 2).

### 8-OH-DPAT challenge

Hypothermia was induced through a subcutaneous injection of 40 µg sterile-filtered 8-OH-DPAT (R-(+)-8-hydroxy-2-(di-n-propylamino)-tetralin; Prod. No. H140; Sigma-Aldrich, St. Louis, USA) delivered in 0.1 ml isotonic saline. Core body temperature was measured rectally immediately prior to the injection using a BAT-12 temperature probe (Physitemp Instruments Inc., Clifton, USA), and HIH was measured at approximate peak hypothermia 30 min following the injection[30]. In between the two measurements each mouse was placed alone in a cage with only bedding, with the exception of Experiment 1, Day 0, where the animals were placed with their cage-mates. The mice were observed during this period for unexpected side effects relating to the injections.

### Experiment 1: Proof-of-Concept

Sixteen male BALB/cAnNTac mice, aged six weeks, weighing approximately 20 g, arrived from the breeder (Taconic, Ry, Denmark), and were subsequently also housed, in two groups of eight (littermates). Prior to the experiment, the animals were acclimatized to the new housing conditions for seven days. On the first day of the experiment (Day 0), four hours after lights on, all the mice were challenged with 8-OH-DPAT. Following a wash-out period of eleven days (Day 11), half of the mice (n = 8) were single-housed for the following 21 days, whereas the other half – acting as a control group – were kept group-housed in the same social setting they came from (n = 8). All animals were left undisturbed with the exception of routine interactions with animal technicians in relation to the changing of feed, water and bedding. The single-housed mice were provided with identical cage enrichments as the group-housed mice. Due to the open cage housing, the isolated mice would still be able to hear and smell their conspecifics throughout the experiment; they were only deprived of direct social contact. No inter-individual aggressions were noted among the group-housed mice during the study. On Day 32, HIH was again measured as previously described.

### Experiment 2: Screening a Population

Male mice on a C57BL/6Pan background were obtained, opportunistically, (at random) from two SPF breeding units and continuously transferred to an experimental unit over a period of two months. The mice, collecting 15 separate substrains (Table 1), were all bred in-house. A total of 215 animals (all destined to be euthanized in the SPF units), of differing ages (Table 2) were challenged. Due to the routine operations in the breeding units some of the males were housed singly (n = 41) whereas others were co-housed with male littermates/female breeders and occasionally with pups. Substrain, age, weight, number of animals in the cage (discounting newborns), cage type and the duration of the single housing, where applicable, were recorded before the animals were challenged. The single-housed mice had been isolated between 11 and 32 days, with a majority of them isolated between two and three weeks (Table 3). All animals were challenged 4–7 h after lights on, when we expected the mice's body temperatures to be at their nadir.

**Table 1:** Substrains collected in Experiment 2.

	Substrain	Single	Group
1.	C57BL/6 (Parent strain)	8	23
2.	C57BL/6-*Prf1^tm^*	-	17
3.	B6.129P2-*Il10^tm^*	-	3
4.	B6.129S7-*Ifngr1^tm^*	10	9
5.	B6.129-*Myd88^tm^*	-	40
6.	B6129F1-*Ifngpfp^tm^*	-	8
7.	B6.129S7-*Ifnab^tm^*	-	6
8.	B6.129-*Ifnb1^tm^*	-	1
9.	B6; 129P2-*Irf7^tm^*	3	33
10.	B6; 129-*Cxcr3cmbkr5^tm^*	-	8
11.	B6129S5-*Gpr39^tm^*	19	23
12.	B6129S5-*Gprc6a^tm^*	1	-
13.	B6129S5-*Rps6ka4^tm^*	-	2
14.	B6BRF1-*Gfp^tm^*	-	1
	Total	41	174

Mice on a C57BL/6 background that were to be euthanized were obtained from two in-house SPF breeding units (laboratory code: Pan), resulting in a random selection of substrains. Substrain information is based on the information available from the breeding units. Due to the opportunistic design of the study, more detailed information on founder lines could not be obtained. The numbering in the left-hand column is used for identifying the substrains in the supporting materials (File S1).

**Table 2:** Summary of data for animals in Experiment 2.

	Group-housed	Single-housed	Test statistics
Age (weeks)	6–40 (Median: 12)	10–49 (Median: 33)	χ^2^_1_ = 34.9, p<0.001
Weight (g)	20–43 (Mean: 30.6)	21–44 (Mean: 32.8)	t_213_ = −3.2, p<0.001
Start temp (°C)	35.6–39.6 (Median: 38.3)	34.6–38.1 (Median: 37.4)	χ^2^_1_ = 52.4, p<0.001

The body weights of the mice conformed to normal distributions and are consequently tested using a Student's t-test. The ages and starting temperatures were not normally distributed and differences between types of housing are tested for using Mann-Whitney U tests.

**Table 3:** Time spent isolated at the time of challenge for the single-housed animals of Experiment 2.

Isolated	Less than 2 weeks	2–3 weeks	3–4 weeks	4–5 weeks
n =	3	23	12	3

## Results

### Experiment 1: Proof-of-Concept

**Figure 1:**
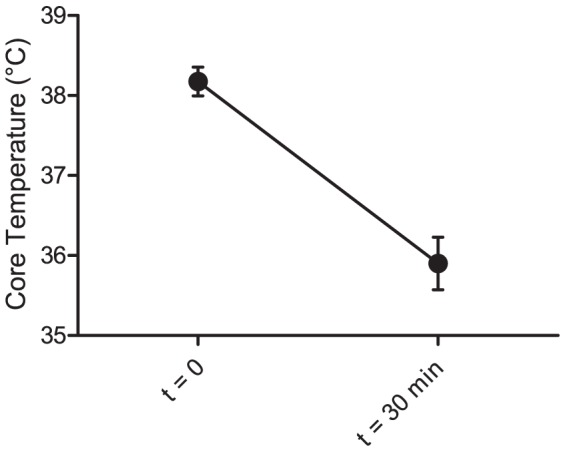
The average drop in body temperature as a response to the 8-OH-DPAT challenge of all 16 BALB/c mice in Experiment 1 before single housing. Error bars represent 95% confidence intervals.

**Figure 2:**
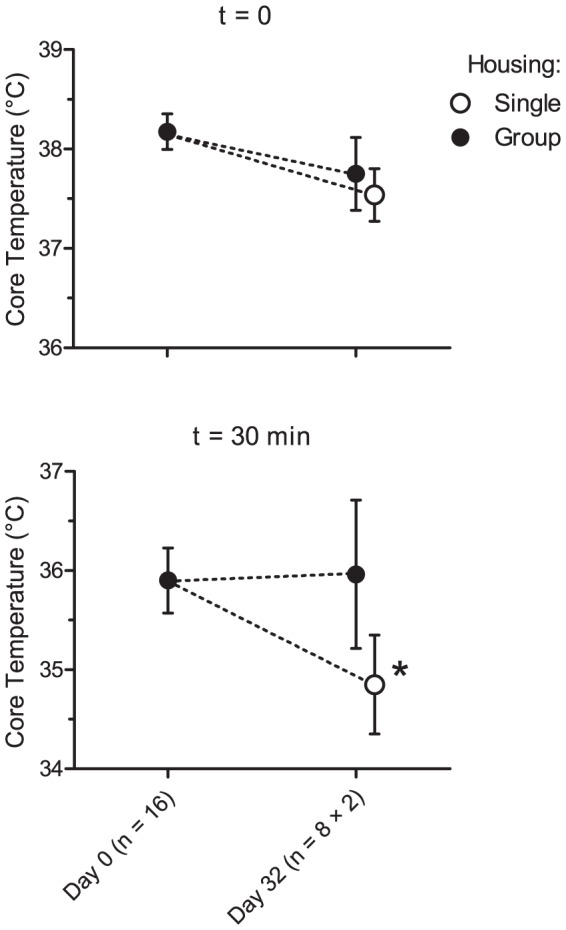
Body temperatures of BALB/c mice at t = 0 and t = 30 min after the 8-OH-DPAT challenge on Day 0 and Day 32 of Experiment 1. Error bars represent 95% CI. The interaction term ‘housing × time’ is significant at the level p<0.05, as represented by the asterisk.

On average the core body temperature of the challenged BALB/c mice decreased by 2.3°C (95% CI: 1.1–3.4°C) in response to the 8-OH-DPAT challenge on Day 0 (Figure 1). On Day 32 the animals responded with differing HIH: Whereas the initial core temperature of the single-housed mice did not differ from that of their group-housed counterparts, the hypothermia was more pronounced in the single-housed mice (Figure 2). Statistical significance was verified using analysis of variance (ANOVA). Housing (Group or Single) and time (Day 0 or Day 32) were offered as explanatory variables to the measured HIH, using the starting temperatures of the mice as covariates. Neither variable could in itself explain the variance in HIH, however the interaction term “housing × time” was found to have a significant effect (F_4,31_ = 3.50, p = 0.015). Being housed singly thus increased the HIH responsiveness in male BALB/c mice over time.

### Experiment 2: Screening a Population

**Figure 3:**
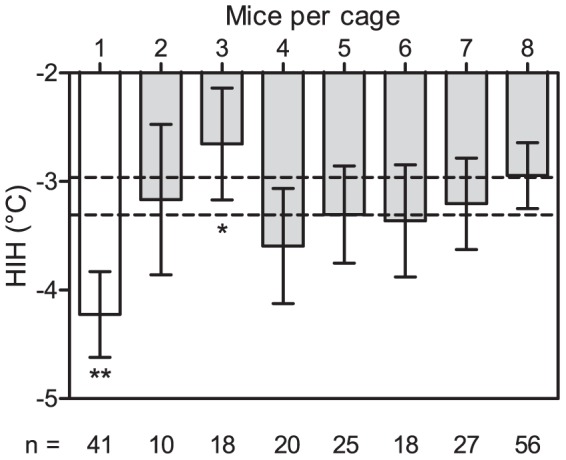
The effect of group composition on HIH found in Experiment 2. The statistical model has been evaluated (estimated marginal means have been calculated) for mice on a C57BL/6 background in different groups weighing 31 grams and with starting temperatures of 38°C (i.e. using population averages). Error bars represent 95% CI of estimates and the dashed lines represent the 95% CI estimate for all group housed mice (2–8). One and two asterisks denote a significant difference from the population mean at the levels p<0.05 and p<0.001, respectively. The numbers of individuals collected in each group (n) are listed below the corresponding bar.

We incorporated all of the challenged C57BL/6 mice into an ANOVA model. No effect on the HIH response was found from the time of day, number of animals to a group, or cage size; these variables were thus excluded from the model. The animals' ages and weights were highly correlated (Pearson's product-moment correlation: p<0.001), which is why we chose to only incorporate the weights in our model rather than using the ages, as this produced a better fit with experimental data. The model confirmed the findings of Experiment 1, with single housed mice on average presenting with a 1.1°C (95% CI: 0.65–1.6) lower core body temperature post-challenge. In addition to a greater hypothermic response, the single-housed mice had, on average, a lower pre-challenge core temperature (Table 2). Although groups of different sizes presented with slight differences in their HIH response (animals housed in groups of three appeared to have a significantly attenuated HIH response), no discernible trend was found for increasing group sizes (Figure 3). For a more descriptive model we then chose to use the time of single housing – ranging from 0 to 32 days – as a covariate rather than treating the type of housing as a binary (single/group) variable. The mice appeared to respond with an increased HIH of 0.5°C for every 10 days of single housing (Table 4), although the effect is likely to be non-linear (after all, the trend will have to bottom-out at some point). In addition, both starting temperature and body weight seemed to affect the end temperature, but to a lesser extent. Both a higher starting temperature and a greater body weight appeared to lead to a greater hypothermic response. To confirm that the difference found in housing did not lie in the different distribution between substrains, data was re-analyzed for only the substrains which had been housed both singly and in groups (refer to Table 1). With the added explanatory variable “substrain”, the effect of housing was still found to be significant (F_1,127_ = 6.1, p<0.05) and to have an effect size of approximately the same magnitude (an average increase in HIH of 0.4°C for every 10 days) as in the preceding model. There was also a significant difference in HIH response between substrains, but the data material is, at current, too small to support speculation on how the genetic differences relate to the results of the HIH challenges. The complete raw data for Experiment 2 can be found in the supporting information (File S1).

**Table 4:** Factors affecting the HIH response in male C57BL/6 mice.

Factor	Effect size (°C)	Test statistics
Body weight (10 g)	0.53 (95% CI: 0.21–0.85)	F_1,214_ = 10.7, p = 0.001
Start temperature (1°C)	0.61 (95% CI: 0.39–0.83)	F_1,214_ = 29.1, p<0.001
Isolation (10 days)	0.51 (95% CI: 0.29–0.73)	F_1,214_ = 21.2, p<0.001

Summary of effects found to influence the outcome of the challenge in Experiment 2 according to an analysis of covariance model (ANCOVA). Incrementing a factor by the value in parenthesis, while all other factors remain constant, is estimated to increase the HIH response by the corresponding effect size; i.e. a mouse housed isolated for an additional 10 days is estimated to increase its HIH in response by, on average, 0.51°C. The linear relation is not expected to hold beyond the extreme bounds of the data material. The effects should therefore not be extrapolated past the minimum/maximum values of the factors presented in Tables 2 and 3.

## Discussion

Based on our findings in two strains of mice, differing in the susceptibility for depressive traits [31], it is concluded that the 8-OH-DPAT-induced hypothermic response differs between single-housed and group-housed male mice. This is, in turn, fairly compelling evidence that being housed alone affects the serotonergic system of the mouse. Furthermore, and perhaps more importantly, the phenomenon is not only inducible in a controlled study, but we find these differences in a randomly chosen genetically heterogeneous population in a mouse breeding unit where males are habitually housed singly, e.g. between matings.

Experiment 2 was, in essence, designed to confirm the findings of Experiment 1 and we deliberately chose different compositions of group-housed mice and single-housed mice isolated for different lengths of time to test the robustness of our initial finding. The opportunistic design of the study provided additional heterogeneity in the tested populations as no one isogenic strain could be obtained in sufficient numbers. However, a strong case has been made for using multiple isogenic strains, rather than a heterogenic population (an outbred stock), if an effect is to be studied across a phenotypically heterogeneous population [32]. That the difference in HIH responsiveness can be found across multiple substrains thus further strengthens the validity of the finding. Although we would ideally have liked the group-housed mice to have been of matching ages and weights as their single housed conspecifics, this was not realizable. Group-housed males in a breeding unit tend to be young littermates that have not yet been mated – once separated from their littermates they will not be re-housed in male-male constellations. Single housing, by contrast, often occurs after matings, which is why the single-housed males were, on average, older. But, although the group averages differed, the population ranges (Table 4) were largely overlapping and consequently the ANCOVA model would not be biased to any greater extent.

A few other phenomena were noted in connection to the 8-OH-DPAT challenges. In addition to housing, the starting temperatures and body weights of the mice had a significant effect on the inducible hypothermia. The former was expected; the inducible hypothermia is brought on through convective heat loss in response to 5-HT_1A_ stimulation in presynaptic neurons [24],[33],[34] (possibly in concert with concomitant 5-HT_1A_ heteroreceptor [35],[36] and 5-HT_7_ activation [37]). This in turn means that the change in temperature will be proportional to the core body temperature at a given point in time, a higher starting temperature enabling a greater reduction in temperature. The HIH response was furthermore positively correlated with the mouse's weight – the heavier the mouse, the greater the hypothermia. This may seem counterintuitive at a glance. Since the injected volume of 8-OH-DPAT was fixed, as opposed to calculated from body weight, the effective dose would decrease (per kg bodyweight) with a bigger mouse. A fatter mouse would, in addition, have more insulating subcutaneous fat that should serve to maintain body temperature. We suspect, however, that the higher metabolic rate of the smaller mice has a key role; the serotonergic agonist being cleared faster and the peak hypothermia occurring faster. With only two points of measurement for an individual it is quite possible that we, for the smaller mice, did not measure the maximum change in body temperature. By recording the rectal temperature repeatedly over time, a better estimate of peak hypothermia could have been established. The procedure, in itself, induces a transient elevation in body temperature (stress-induced hyperthermia), however, which counteracts the 5-HT_1A_-inducible hypothermia [38]. By implanting telemetric temperature monitors, the HIH of mice of differing weights could more accurately have been compared (as has been used to great effect in other studies of hypothermic responses to serotonergic agonists [38],[39]), but in the present setup, the required surgical procedure and post-operative recovery would most likely have influenced the mice's physiological and emotional states, plausibly overriding any effects of single-housing. In addition to having a greater hypothermic response to 8-OH-DPAT the singe-housed mice of Experiment 2 also, on average, had a lower starting temperature. Similar findings have been noted in other contexts. Single-housed mice tend, for example, to prefer higher temperatures than their group-housed conspecifics by approximately 1°C [40] and Späni and collaborators observed, similarly to the present study, that a cohort of single-housed male mice (outbred NMRI stock) had significantly lower body temperatures than did their group-housed counterparts. The latter study furthermore concluded that the difference could not be explained through differing activity levels [41]. The present study, and others studying core body temperatures in mice [42], have not found a trend between the number of group-housed mice to a cage and their body temperatures (Figure 3); we can therefore speculate that huddling did not have an influence on the temperatures. Mice huddle together to minimize energy expenditure in their inactive period [43], but at the studied temperature and with the nest-building materials provided this huddling is probably used to lower the metabolic rate rather than to maintain body temperature [44]. With this in mind, we tentatively suggest that the effect on the serotonergic system brought on by single housing not only affects the hypothermic response in the 8-OH-DPAT challenge, but may also influence core body temperature. Temperature dysregulation is not uncommon with (human) depressions [45],[46] and is a known side effect with drugs targeting the serotonergic system, e.g. selective serotonin reuptake inhibitor (SSRI) drugs [47]. Antidepressant compounds are extensively screened in mice in drug development [48], where differential 5-HT receptor expressions or activities may have profound consequences. It has for example been shown that the antidepressant effect of the SSRI fluoxetine in male C57BL/6 mice is modulated by the animals' housing conditions [49]. In fact, there are findings dating back more than four decades testifying to the fact that single housing may modulate the effect of neuroactive compounds (e.g. [50]). Inadvertently inducing depressive states in a subset of laboratory mice is of course a welfare concern, but it moreover influences experimental results. With a currently poor translational success rate for development of neuroleptics from animal studies to effect in humans [51],[52], refining/controlling housing and husbandry conditions for the tested laboratory mice is essential (“envirotyping”) [53],[54]. When evaluating and improving ambient (biotic/abiotic) conditions we propose that the 8-OH-DPAT challenge may serve as a simple, but powerful, tool in assessing the serotonergic states of laboratory mice.

In closing, it is clear that male mice will not only choose social contact when given the option, but will also, when it is deprived, be negatively affected by its absence. The present study further suggests that the 8-OH-DPAT challenge may be a useful tool for assessing the effect of this social deprivation on the serotonergic state of male laboratory mice. Whereas the method requires, and merits, additional study, it potentially allows not only for an unbiased, biochemical evaluation of sub-acute stressors, but potentially allows for determining whether these detrimental effects can be counteracted.

## Supporting Information

File S1 **Complete raw data set for Experiment 2.** Listed information is the type of housing, cage occupancy and length of single housing (in days); the age (in weeks), body weight and substrain (refer to Table 1) of the animal; and the core body temperatures before challenge, at peak hypothermia and the resulting HIH.
(CSV)
